# Supplementary material for: Expression Profile of miR-199a and Its Role in the Regulation of Intestinal Inflammation
Source: Animals (Basel). 2023 Jun 14;13(12):1979. doi: 10.3390/ani13121979 (PMC10294982; doi:10.3390/ani13121979)
Supplement: Supplementary file 1 [file animals-13-01979-s001.zip › Supplementary Figures.pdf]

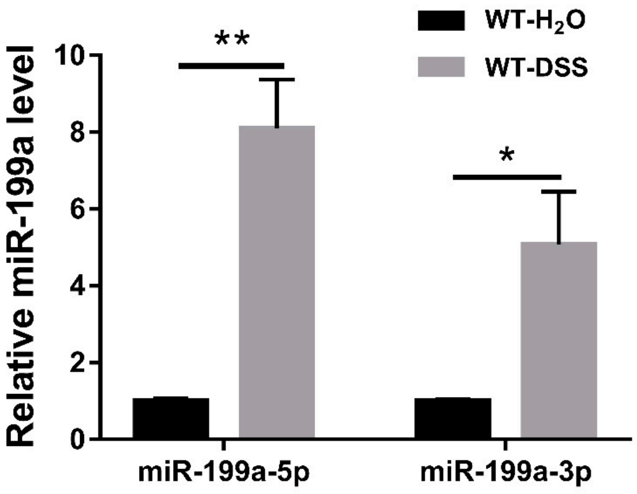

Supplementary Figure S1. Changes in miR-199a-5p/3p expression in WT mice with DSS treatment (n = 4/group; *, $P < 0.05$; **, $P < 0.01$).

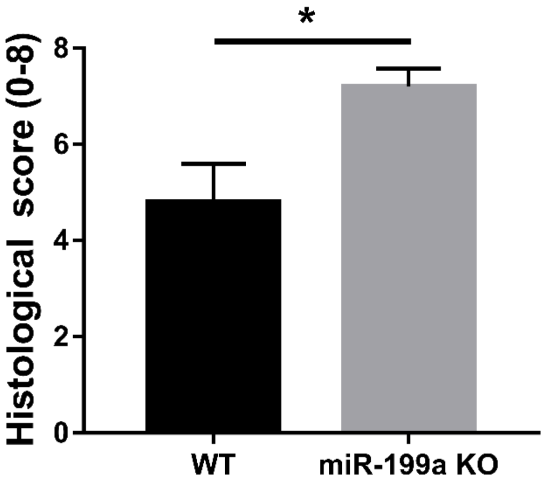

Supplementary Figure S2. Histopathological score of colonic inflammation (n = 5/group; *, $P < 0.05$).

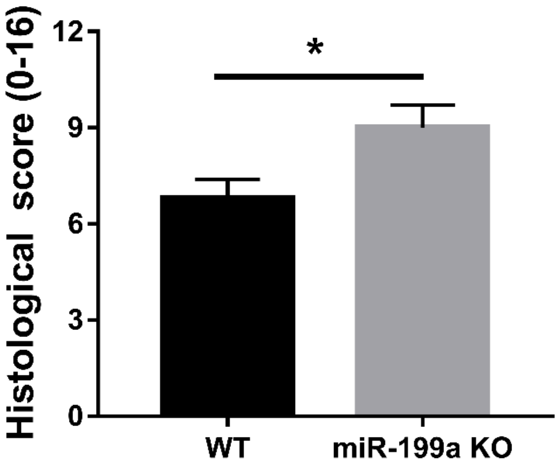

Supplementary Figure S3. Histopathological score of splenic inflammation (n = 5/group; *, $P < 0.05$).

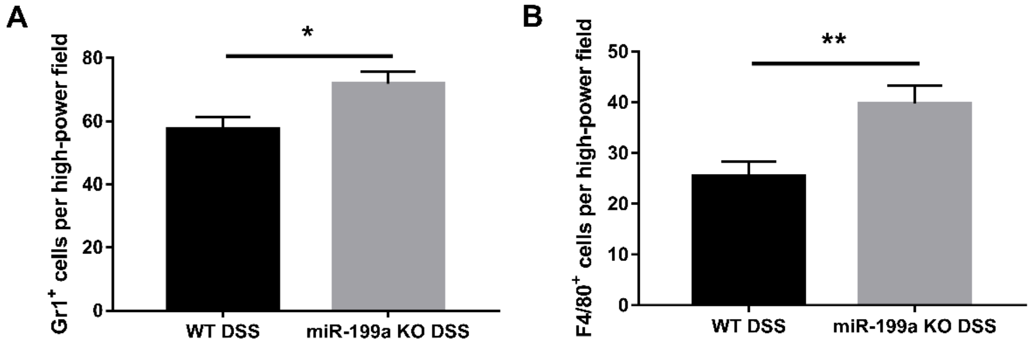

Supplementary Figure S4. $Gr1^+$ neutrophils and $F4/80^+$ macrophages in colonic tissues. $Gr1^+$ and $F4/80^+$ cells were quantified by counting four high-power fields on each section (n = 12/group; *, $P < 0.05$; **, $P < 0.01$).
