# Supplementary material for: Expression Profile of miR-199a and Its Role in the Regulation of Intestinal Inflammation
Source: Animals (Basel). 2023 Jun 14;13(12):1979. doi: 10.3390/ani13121979 (PMC10294982; doi:10.3390/ani13121979)
Supplement: Supplementary file 1 [file animals-13-01979-s001.zip › Table S1.pdf]

Table S1. Histological scoring system for splenic inflammation.

Score	Yes/⇑⇓/Severity Grade*	No
Periarteriolar lymphoid sheath		
Increased/decreased size		
Increased/decreased number		
Increased/decreased lymphocytes		
Marginal zone		
Increased/decreased size		
Increased/decreased lymphocytes		
Follicles		
Increased/decreased numbers		
Increased/decreased lymphocytes		
Increased/decreased germinal centers		
Red pulp		
Increased/decreased size		
Increased/decreased lymphocytes		
Increased hematopoietic cells		

*One recommendation for a grading scheme would be 0 = normal, 1 = minimal, 2 = mild, 3 = moderate, 4 = marked.
