# Supplementary figures and images for: Expression Profile of miR-199a and Its Role in the Regulation of Intestinal Inflammation
Source: Animals (Basel). 2023 Jun 14;13(12):1979. doi: 10.3390/ani13121979 (PMC10294982; doi:10.3390/ani13121979)

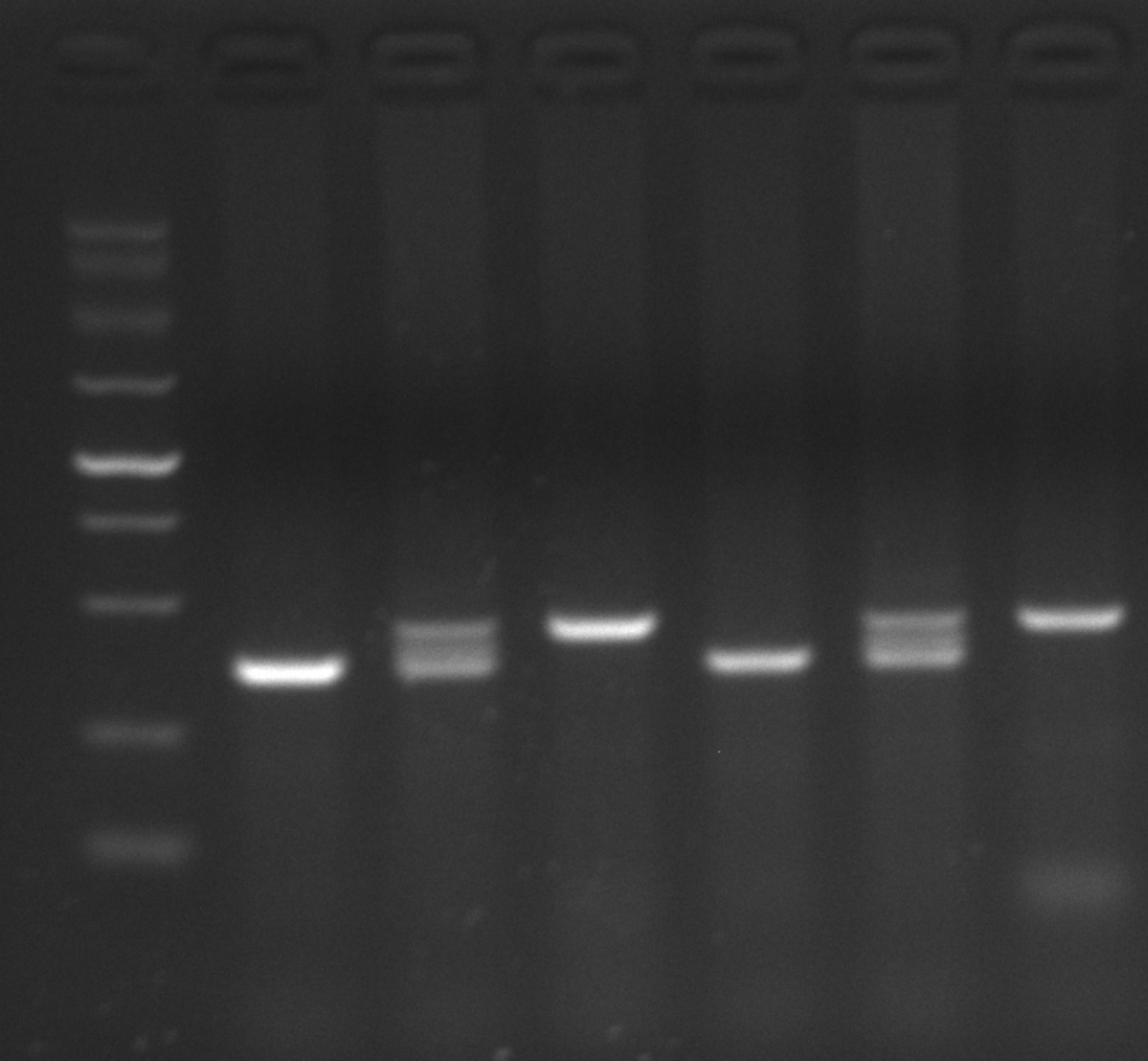

Supplement: Supplementary file 1 [file animals-13-01979-s001.zip › animals-2414223-original gel electrophoresis figures.pdf]
